# Pediatric Emergency Medicine Didactics and Simulation: JumpSTART Secondary Triage for Mass Casualty Incidents

**DOI:** 10.7759/cureus.40009

**Published:** 2023-06-05

**Authors:** Yongtian Tina Tan, Cassandra Koid Jia Shin, Brian Park, Anita Bharath, Robyn Wing, Cecilia Monteilh, Elizabeth Sanseau, Brittany Boswell, Jean I Pearce, Maureen Luetje, Brianna Enriquez, Mark Cicero, Anita Thomas

**Affiliations:** 1 Pediatric Emergency Medicine, Seattle Children's Hospital, Seattle, USA; 2 Pediatric Emergency Medicine, University of Arizona College of Medicine, Phoenix, USA; 3 Pediatric Emergency Medicine, Hasbro Children's Hospital, Providence, USA; 4 Pediatric Emergency Medicine, Children's Hospital of Philadelphia, Philadelphia, USA; 5 Pediatric Emergency Medicine, Stanford University School of Medicine, Palo Alto, USA; 6 Pediatric Emergency Medicine, Medical College of Wisconsin, Milwaukee, USA; 7 Pediatric Emergency Medicine, Yale School of Medicine, New Haven, USA

**Keywords:** triage, emergency medicine, disaster response and preparedness, secondary triage, pediatric emergency department, medical education and simulation, triage protocols, mass casualty incident

## Abstract

Mass casualty incidents (MCI), particularly involving pediatric patients, are high-risk, low-frequency occurrences that require exceptional emergency arrangements and advanced preparation. In the aftermath of an MCI, it is essential for medical personnel to accurately and promptly triage patients according to their acuity and urgency for care. As first responders bring patients from the field to the hospital, medical personnel are responsible for prompt secondary triage of these patients to appropriately delegate hospital resources. The JumpSTART triage algorithm (a variation of the Simple Triage and Rapid Treatment, or START, triage system) was originally designed for prehospital triage by prehospital providers but can also be used for secondary triage in the emergency department setting. This technical report describes a novel simulation-based curriculum for pediatric emergency medicine residents, fellows, and attendings involving the secondary triage of patients in the aftermath of an MCI in the emergency department. This curriculum highlights the importance of the JumpSTART triage algorithm and how to effectively implement it in the MCI setting.

## Introduction

A mass casualty incident (MCI) is defined by the Pan American Health Organization (PAHO) "as an event which generates more patients at one time than locally available resources can manage using routine procedures. It requires exceptional emergency arrangements and additional or extraordinary assistance [1]." MCIs are provoked by a disaster (natural or human-made) [2]. MCIs disproportionately affect children, a population known to be particularly vulnerable due to anatomic, physiologic, immunologic, developmental, and psychological factors [3]. In the chaos of an MCI, especially when children are involved, it is crucial for first responders to accurately triage, or "sort patients according to the urgency of their need for care [4]." Defined MCI protocols specific to pediatrics are important given this group’s vulnerability associated with higher morbidity and mortality in the setting of an MCI [5]. Two prevalent pediatric triage algorithms include JumpSTART (Simple Triage and Rapid Transport), which is the most commonly used algorithm, and SALT (Sort, Assess, Lifesaving Interventions, Treat/Transport). While research suggests these two systems appear similar in accuracy and ease of use, in pediatric simulated MCI, the JumpSTART approach averaged eight seconds faster per patient in time to triage designations (the average time to triage per patient using JumpSTART among study participants was 26 seconds compared to 34 seconds using SALT) [6].

Mass casualty incidents are becoming increasingly common [7], yet sporadic occurrences and limited resources add to the challenge of readiness for emergency departments (EDs). Emergency medicine is often at the forefront of responding to MCI, and responding effectively is crucial to minimizing the morbidity and mortality of patients while maximizing available resources. While simulation cases that address MCI triage have been published [8, 9], to our knowledge, one focused on pediatric MCI triage does not yet exist. We aim to use this simulation to teach the principles of the JumpSTART algorithm in the emergency department setting, to better understand the triage initially performed by prehospital professionals in the field, and to put into practice a novel application of this tool for secondary triage in the hospital. We hope that through this simulation, we can: 1) improve familiarity with JumpSTART for pediatric emergency medicine physicians (PEM) via didactics, and 2) provide PEM physicians an opportunity to utilize the JumpSTART triage to categorize patients prior to a real-life MCI or disaster.

## Technical report

Methods

Case Overview

This simulation case was developed by pediatric emergency medicine (PEM) physicians with expertise in curriculum development, simulation, and disaster medicine. The scenario was fictional but based on a realistic potential natural disaster situation surrounding the arrival of patients to the emergency department immediately after an 8.2 Richter scale earthquake. Fifteen victim scenarios ranging from ages six months to 62 years old were presented as part of the simulation, and participants were asked to triage these patients according to the JumpSTART and START algorithms. A simplified simulation using only patient description cards was trialed on ten participants consisting of two medical students, two pediatric residents, and six pediatric emergency fellows initially at Seattle Children’s Hospital prior to rolling out to the larger group. Feedback from the initial simulation resulted in modifications to the survey as well as design improvements, including the addition of manikins and embedded participants (EPs) within the case. The final simulation was implemented with residents, fellows, and attendings at four institutions as part of their routine education curriculum. No prerequisite knowledge was necessary to participate.

Participants

We implemented this simulation with a total of 32 participants, including 20 pediatric emergency medicine fellows, eight pediatric emergency attending physicians, two advanced practitioners, and two pediatric residents, across four training sites over a six-month period. Participants had prior experience with simulation and medical resuscitation. Participants were oriented to the low-fidelity manikins, cards with patient descriptions (when not enough manikins were available), and EPs prior to the case. Each site conducted the simulation once.

Personnel

The facilitators were pediatric emergency medicine attending physicians. The sessions were ideally run with at least two facilitators, but given resource limitations, they could and were at times run with one facilitator. As no high-technology manikins were utilized in the simulation, no simulation technician was required.

Setting and Equipment

The setting is the pediatric emergency department, and the simulation can be conducted in situ or in a simulation lab or classroom. A separate space for debriefing can be used if necessary. The cases can be modified depending on the availability of low- and high-fidelity manikins; embedded participants (EPs) can also be used if available. Moulage can be applied to manikins and EPs using inexpensive makeup. The minimum required materials include the JumpSTART victim descriptions and case summary, the JumpSTART triage worksheet, and the JumpSTART triage answer key (Appendices 1-3).

Environmental Preparation

We recommend arriving at least 30 minutes prior to participants' arrival in order to set up. Place 15 individual "victim" cards and/or manikins and EPs around the room. High- or low-fidelity manikins or EPs may be used, depending on institutional resources. Please refer to the JumpSTART triage victim guide (Appendix 4) for recommendations on which victim cases may be appropriate for high fidelity and EPs and an example set-up (Appendix 5).

Each participant is given a JumpSTART triage worksheet (Appendix 2) to fill out during the simulation. Depending on the size of the simulation space and the number of participants, one may consider dividing into smaller groups; some sites found five participants at a time to be a reasonable number. Each participant should be given the full 10 minutes to go around the room, perform triage, and fill out the worksheet.

Prebriefing

We recommend a prebrief be completed outside of the simulated space to avoid distraction and to set the stage for less experienced learners to have some background on JumpSTART triage; we recommend allotting at least ten minutes for this portion. We suggest utilizing the didactics (Appendices 6-14) here [10-14]. We recommend a mental health disclaimer in light of recent occurrences of gun violence and mass casualty incidents. The standard prebrief should also include the simulation learning contract, an overall orientation to the room set-up, and expectation setting. The facilitator can then read the stem of a mass casualty incident related to an earthquake (Appendix 1) to the participants. We recommend giving participants at least three minutes to review the adult START and pediatric JumpSTART triage algorithms prior to starting the simulation. They may have copies of the algorithms (Appendix 14) as a reference as they move through the simulation.

Debriefing

The guidelines available in Appendix 15 are used to facilitate debriefing sessions after the simulation. This debriefing guide is adapted from another simulation curriculum at Seattle Children’s Hospital as well as the PEARLS (Promoting Excellence and Reflective Learning in Simulation) Debriefing Framework [15, 16]. This tool allows each facilitator to tailor the discussion based on the needs and performance of the participants. We recommend beginning the debrief by allowing participants to provide general reflections on their experiences. The facilitator can use observations made by participants and facilitators as lead points into discussions on the triage process for each individual case and on the overall process. We recommend setting aside at least 10 to 15 minutes for the debrief.

Results

Participants completed a post-simulation survey following the debrief (Appendix 16). They were asked to state their agreement with evaluative statements using the Likert scale (Table 1).

**Table 1:** Participants’ experience during the simulation session and clinical confidence after the session (Likert scale: 1=strongly disagree, 3=neutral, 5=strongly agree); N=32

Participant survey question	Mean Likert score	Range	Standard deviation
This simulation is relevant to my work	4.9	4-5	0.25
This simulation was realistic	4.2	2-5	0.79
This simulation was effective in teaching JumpSTART triage skills	4.9	4-5	0.30
I feel prepared to efficiently use JumpSTART triage in secondary triage of pediatric MCI patients	4.4	3-5	0.67
I feel confident correctly categorizing pediatric MCI victims using the JumpSTART triage algorithm	4.3	3-5	0.60
This simulation created a safe environment	4.9	4-5	0.25
This simulation promoted reflection and team discussion	4.9	4-5	0.25

They were surveyed about their experience during the educational session and about their clinical confidence related to the learning objectives after participating in the session. They were invited to answer free-response questions related to their experience (Table 2).

**Table 2:** Participants’ comments and feedback after participating in the simulation

Implementation site	Participant comments
Site #1	Participants appreciated the opportunity to think systematically using the JumpSTART algorithm in a mass casualty scenario. They reported gaining familiarity with the key components of the algorithm and appreciated the importance of performing rapid assessments.
Site #2	Participants commented on the value of debriefing on each individual scenario at the end of the simulation. They reported a better understanding of key differentiators between patient triage categories, especially how to appropriately distinguish between "yellow" and "red" category patients.
Site #3	There was an appreciation for learning how to remove the emotional aspect of the triage process in a highly tense situation. Participants also expressed an improved grasp of how secondary triage differs for patients based on age. One reported "retaining mindset regarding who to immediately intervene on, taking emotional aspects out of the thought process to use the algorithm."
Site #4	Participants felt that the simulation created a safe environment for them to develop high-level assessment and problem-solving skills in a mass casualty scenario. Many reported having more confidence in the triage approach should they encounter real-life MCIs in the future. One participant wrote, "This is helpful for learning the algorithm in a safe environment as it is much different from how I typically think of triaging patients."

The curriculum and simulation received strong positive feedback from participants. There was a good representation of the differing levels of simulation fidelity used across sites, with most sites using a mix of EPs, manikins, and paper victim cards (Table 3).

**Table 3:** Breakdown of participant types and use of manikins, embedded participants, and paper scenarios at each implementation site PEM: pediatric emergency medicine; EPs: embedded participants.

Implementation site	Participants	Breakdown of manikins, EPs, and paper scenarios
Site #1	6 (5 PEM fellows, 1 pediatric resident)	4 manikins, 4 EPs, and 7 paper scenarios
Site #2	5 (3 PEM fellows, 2 PEM attendings)	15 paper scenarios
Site #3	10 (7 PEM fellows, 2 PEM attendings, and 1 pediatric resident)	3 manikins, 3 EPs, and 9 paper scenarios
Site #4	11 (5 PEM fellows, 4 PEM attendings, and 2 advanced practitioners)	4 manikins, 2 EPs, and 9 paper scenarios

## Discussion

The process and principles of secondary triage in a mass casualty incident differ significantly from the standard approach to patient triage in the emergency department setting. The JumpSTART algorithm creates a systematic framework for medical professionals to effectively and appropriately prioritize resources in a disaster setting for pediatric patients. This curriculum gave participants an opportunity to build confidence and mental preparation should they ever encounter a real-life MCI disaster involving adults and children and improve their understanding of the triage done by first responders during MCIs. Feedback from our cohort of participants was overwhelmingly positive in terms of simulation experience and clinical confidence. We designed the curriculum to be adaptable for different resource settings in terms of the availability of simulation equipment and personnel, as well as for ease of implementation. It was designed for advanced learners who may work in an emergency setting, including medical students, residents, advanced care providers, fellows, and attendings. That being said, this would be useful for any person who might find themselves in the role of a first responder in an MCI, including but not limited to trainees and professionals practicing in emergency medical services, nursing, surgery, anesthesia, pediatrics, family medicine, and emergency medicine.

We performed iterative improvement by first trialing a simplified, exclusively paper scenario version with a small group of PEM fellows and pediatric residents. Feedback from this trial was used to finalize our cases and surveys as well as add embedded participants and manikins for the final rollout. One of our earliest trials erroneously used SALT in their simulation, which directed us to troubleshoot and emphasize for future iterations that using JumpSTART was the main objective and preferred method of triage for the specific purposes of this simulation. Feedback specific to each victim scenario was collected and incorporated with each iteration; for example, originally, victim three was described as "pulseless", which did not correlate with other symptoms such as withdrawing from stimuli due to pain. His prompt was edited to have a weak radial pulse instead. Additionally, we found timing as well as a mental health disclaimer or trigger warning to be key to running this simulation. For instance, one pilot site implemented this simulation a few weeks after a national mass shooting, and participants were receptive to the trigger warning included in the prebrief regarding the potential to bring up past or recent trauma related to the recent mass shooting. This also invited an open discussion of the emotional aspects of the triage process in the debrief. The importance of debriefing after traumatic events in the clinical setting has been well described and shared in the literature [17].

We acknowledge the limitations of using the Likert scale as the main method of assessment for this simulation. There is a risk of agreement bias in this form of measurement, and assessing participants’ feelings of confidence does not fully reflect actual competency and internalization of concepts. For future improvement, it would be helpful to implement a pre- and post-simulation competency assessment to measure gains in knowledge. We also recognize the opportunity to improve the realistic aspect of simulation by incorporating more embedded participants and advanced manikins that require participants to actively obtain information on their respiratory effort, perfusion, and mental status rather than having this data provided on paper. Future iterations of this simulation can also incorporate real-life teamwork aspects of MCI triage, allowing participants to collaborate with one another in the process and involving other medical team members such as nurses and respiratory therapists. Finally, while JumpSTART triage uses the color black to denote expectant management, we propose changing to the color blue for this code, as many organizations such as Advanced Trauma Life Support have done [18], to remove racial connotations.

## Conclusions

This simulation curriculum was designed to create a safe and structured environment for participants to learn and put into practice the JumpSTART triage algorithm in a low-frequency, high-impact mass casualty scenario involving children. Using JumpSTART for secondary triage in the hospital is a novel concept not previously described in the literature. The simulation can be easily implemented with varying degrees of fidelity and expanded to include trainees and professionals practicing in a variety of settings within and outside of emergency medicine. This technical report provides facilitators with the materials required to implement the simulation to better prepare their teams for future real-life MCIs and disasters.

## Figures and Tables

**Figure 1:**
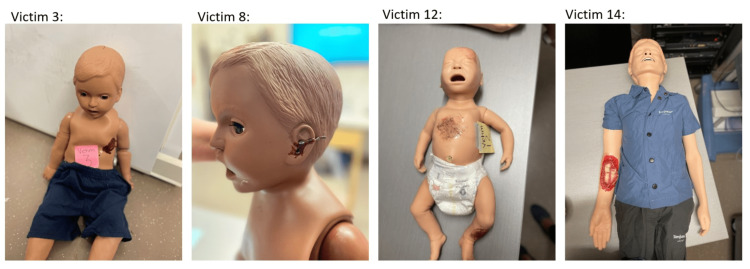
JumpSTART example set-up

**Figure 2:**
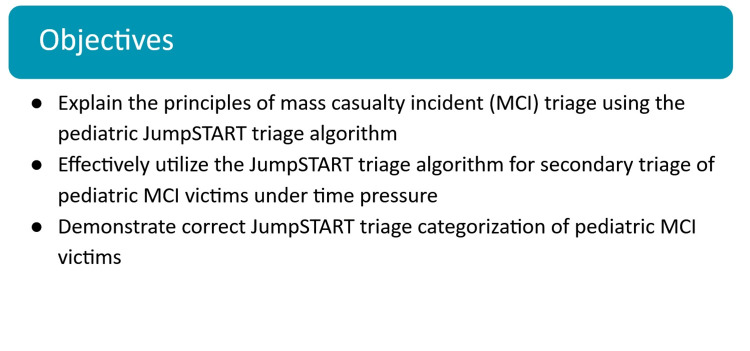
Slide 1

**Figure 3:**
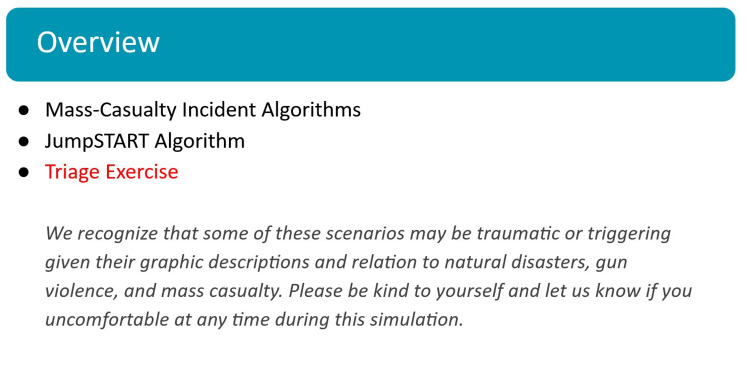
Slide 2

**Figure 4:**
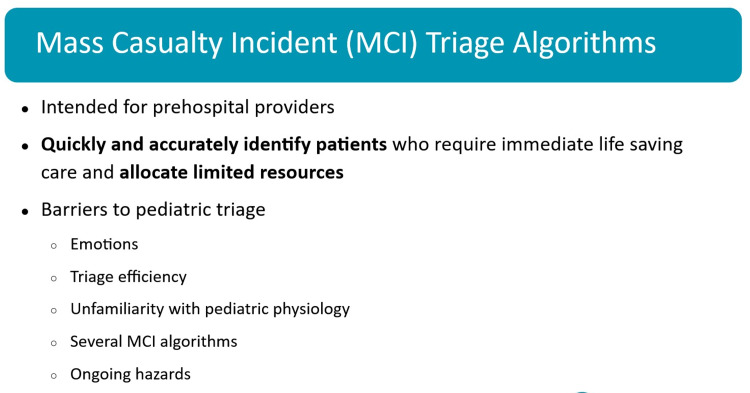
Slide 3

**Figure 5:**
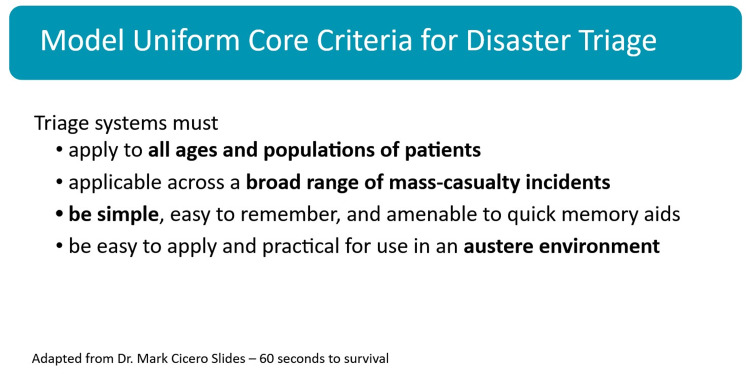
Slide 4

**Figure 6:**
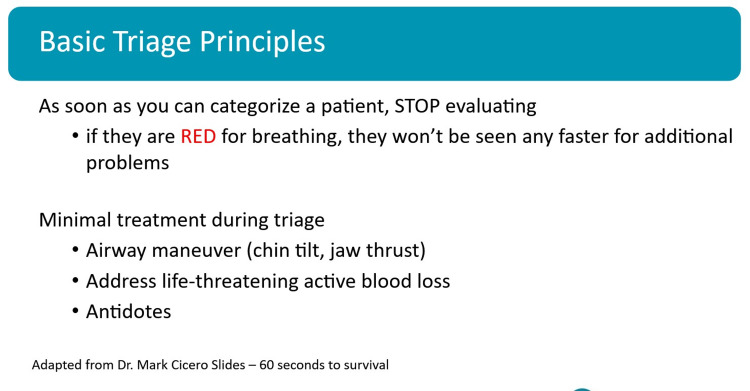
Slide 5

**Figure 7:**
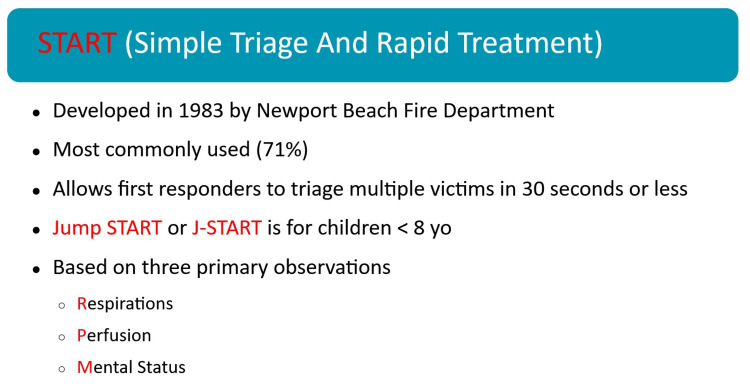
Slide 6

**Figure 8:**
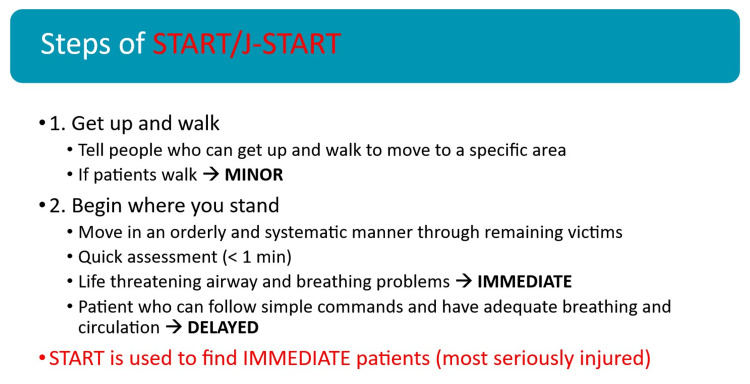
Slide 7

**Figure 9:**
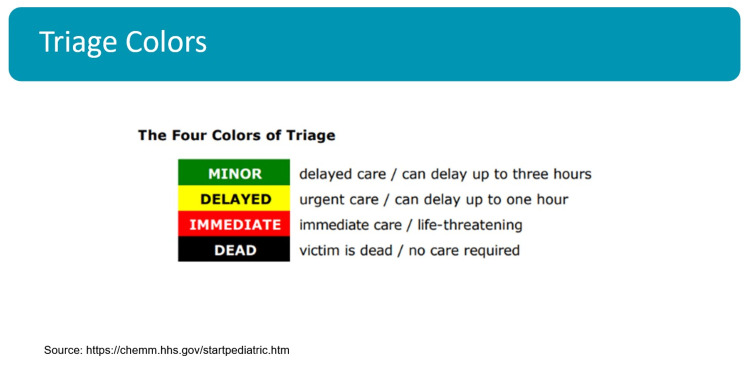
Slide 8

**Figure 10:**
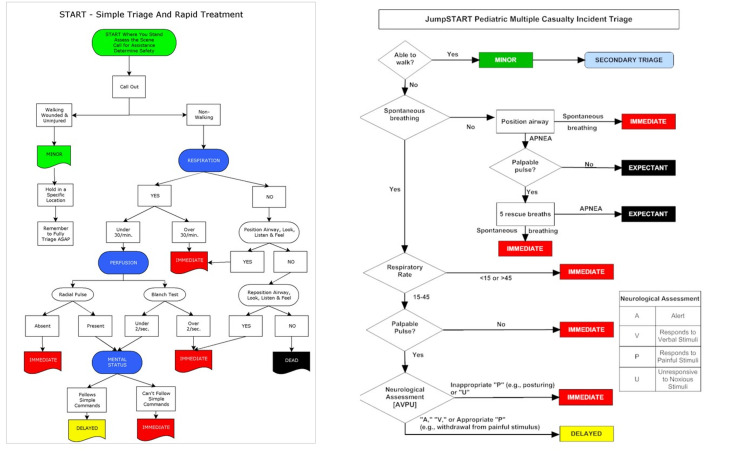
Slide 9

**Figure 11:**
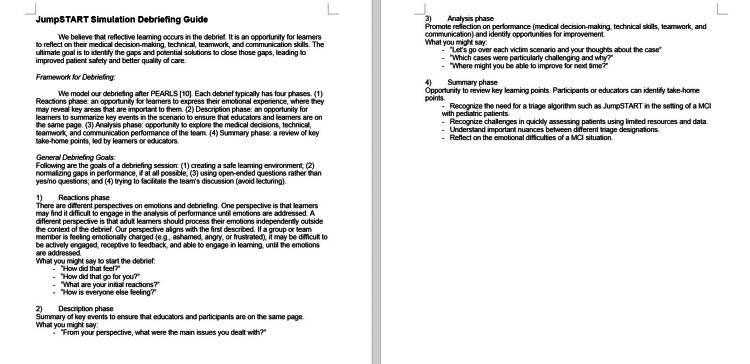
JumpSTART debriefing guide

**Figure 12:**
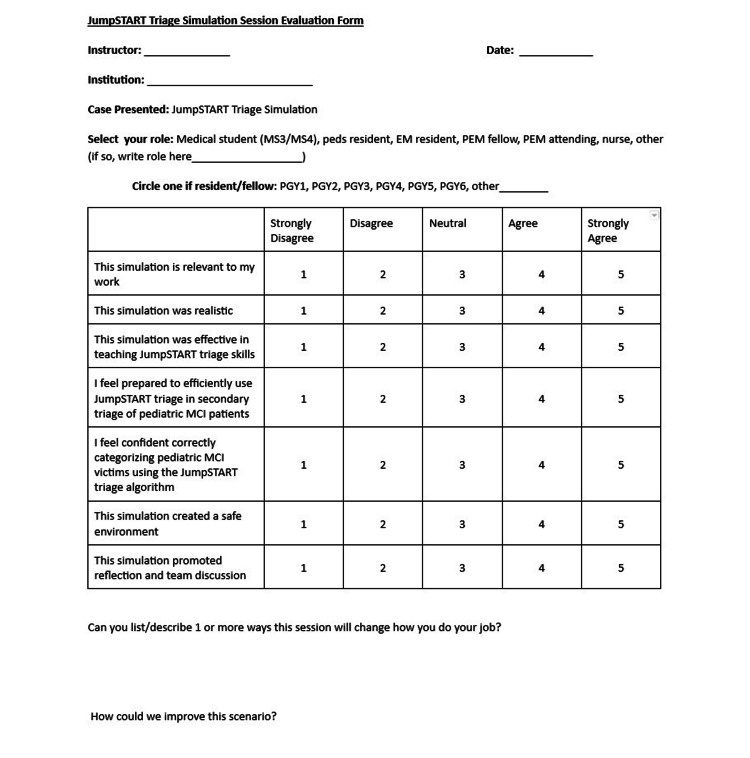
JumpSTART triage simulation evaluation form

## Appendices

Appendix 1

**Table 4:** JumpSTART triage victim descriptions and case summary yo: year-old; mo: month-old; F: female; M: male; RR: respiratory rate

Case Summary: "You are working in the ED when you feel the ground shaking. There has been an 8.2 Richter-scale earthquake. After checking yourself and your surroundings for safety, you confirm that your staff and your ED patients are okay and that the ED is still standing. However, new patients begin streaming into the ED. You have been designated as a hospital triage officer to sort this influx of patients for priority medical treatment in the ED. Some of these patients have been triaged in the field, but it’s important to reassess the patient and perform a secondary triage designation upon their arrival in the ED. You have 10 minutes to triage all patients."					
Victim (age in years, sex)		RR	Perfusion	Mental status	Other
1	7-yo F	10	Distal pulse present	Groans in response to painful stimuli	Found lying down, carried in by a bystander
2	45-yo M	0	Pulseless	Unresponsive	Trapped under a beam
3	4-yo M	40	Weak radial pulse	Withdraws from painful stimuli	Arm deformity, sucking chest wound
4	18-mo F	35	Distal pulse present	Crying	Limping, abrasions, and some embedded gravel
5	5-yo M	20	Distal pulse present	Obeys commands	Complains that they cannot move or feel their legs.
6	30-yo F	18	Distal pulse present	Obeys commands	Sitting on the side of the road, she reportedly ambulated there, clutching her head c/o dizziness.
7	25-yo F	12	CR>4 sec	Eye movement in response to stimuli, not speaking	Appears to be six months pregnant.
8	2-yo M	28	Distal pulse present	Not following commands	Sitting on the shoulder of the road, blood in ears, unwilling to walk
9	12-yo F	8	Pulse absent	Unresponsive	Impaled by a wooden beam
10	17-yo F	0	Weak radial pulse	Unresponsive	Trapped under rubble, apneic after five rescue breaths
11	62-yo M	28	CR<2	Crying for help, able to recall events	Leg caught under rubble, open fracture
12	6-mo M	0	Absent pulse	Moaning initially, now unresponsive	Was found down on the ground with abrasions all over his body and a large occipital hematoma.
13	8-yo F	40	Weak radial pulse	Responds to verbal stimuli	Large bruise forming on the abdomen, abrasions on extremities, unable to walk
14	13-yo M	48	Rapid and weak	Blank stare	Partial amputation of the right arm, diaphoretic
15	3-yo F	36	Bounding pulse	Alert but won’t speak	Abrasions to neck and torso; lacerations on arms with some embedded glass, refusing to move legs

Appendix 2 

**Table 5:** JumpSTART triage worksheet

Victim	Triage category (circle)	Notes
1	RED YELLOW GREEN BLACK	
2	RED YELLOW GREEN BLACK	
3	RED YELLOW GREEN BLACK	
4	RED YELLOW GREEN BLACK	
5	RED YELLOW GREEN BLACK	
6	RED YELLOW GREEN BLACK	
7	RED YELLOW GREEN BLACK	
8	RED YELLOW GREEN BLACK	
9	RED YELLOW GREEN BLACK	
10	RED YELLOW GREEN BLACK	
11	RED YELLOW GREEN BLACK	
12	RED YELLOW GREEN BLACK	
13	RED YELLOW GREEN BLACK	
14	RED YELLOW GREEN BLACK	
15	RED YELLOW GREEN BLACK	

Appendix 3

**Table 6:** JumpSTART triage answer sheet yo: year-old; mo: month-old; F: female; M: male; RR: respiratory rate

Victim (age in years and sex)		RR	Perfusion	Mental status	Other	Triage category
1	7-yo F	10	Distal pulse present	Groans in response to painful stimuli	Found lying down, carried in by a bystander	RED Immediate
2	45-yo M	0	Pulseless	Unresponsive	Trapped under a beam	BLACK Expectant/deceased
3	4-yo M	40	Weak radial pulse	Withdraws from painful stimuli	Arm deformity, sucking chest wound	RED Immediate
4	18-mo F	35	Distal pulse present	Crying	Limping, abrasions, and some embedded gravel	GREEN Minor
5	5-yo M	20	Distal pulse present	Obeys commands	Complains that they cannot move or feel their legs.	YELLOW Delayed
6	30-yo F	18	Distal pulse present	Obeys commands	Sitting on the side of the road, she reportedly ambulated there, clutching her head c/o because of dizziness.	GREEN Minor
7	25-yo F	12	CR>4 sec	Eye movement in response to stimuli, not speaking	Appears to be six months pregnant	RED Immediate
8	2-yo M	28	Distal pulse present	Not following commands	Sitting on the shoulder of the road, blood in the ears, unwilling to walk	RED Immediate
9	12-yo F	8	Pulse absent	Unresponsive	Impaled by a wooden beam	RED Immediate
10	17-yo F	0	Weak radial pulse	Unresponsive	Trapped under rubble, apneic after five rescue breaths	BLACK Expectant/deceased
11	62-yo M	28	CR<2	Crying for help, able to recall events	Leg caught under rubble, open fracture	YELLOW Delayed
12	6-mo M	0	Absent pulse	Moaning initially, now unresponsive	Was found down on the ground with abrasions all over the body, a large occipital hematoma	BLACK Expectant/deceased
13	8-yo F	40	Weak radial pulse	Responds to verbal stimuli	Large bruise forming on the abdomen, abrasions on extremities, unable to walk	YELLOW Delayed
14	13-yo M	48	Rapid and weak	Blank stare	Partial amputation of the right arm, diaphoretic	RED Immediate
15	3-yo F	36	Bounding pulse	Alert but won’t speak	Abrasions to neck/torso, lacerations on arms with some embedded glass, refusing to move legs	YELLOW Delayed

Appendix 4

**Table 7:** JumpSTART triage victim guide yo: year-old; mo: month-old; F: female; M: male; RR: respiratory rate

Victim #	Victim (age in years and sex)	RR	Perfusion	Mental status	Ambulatory	Details	Triage category	Moulage tips	Paper appropriate	Manikin appropriate	Embedded participant appropriate
1	7-yo F	10	Distal pulse present	Groans in response to painful stimuli	No	Found lying down, carried in by a bystander	RED		Yes	Junior size	Yes
2	45-yo M	0	Pulseless	Unresponsive	No	Trapped under a beam	BLACK	Position the beam on top of the patient	Yes	Adult size	
3	4-yo M	40	Weak radial pulse	Withdraws from painful stimuli	No	Arm deformity, sucking chest wound	RED	Bruising on chest, wedge on the forearm	Yes	Junior size	
4	18-mo F	35	Distal pulse present	Crying	Yes	Limping, abrasions, and some embedded gravel	GREEN	Dirt, gravel, red (abrasions) markings on lower extremities, dirt on arms/face	Yes	Toddler size	
5	5-yo M	20	Distal pulse present	Obeys commands	No	Complains that they cannot move or feel their legs.	YELLOW	Sitting on the ground, legs covered in dirt. Scared and anxious. Unable to move legs or get up	Yes	Toddler size	
6	30-yo F	18	Distal pulse present	Obeys commands	Yes	Sitting on the side of the road, she reportedly ambulated there, clutching her head because of dizziness.	GREEN	Abrasions on the forehead	Yes		Yes
7	25-yo F	12	Cap refill > 4 sec	Eye movement in response to stimuli, not speaking	No	Appears to be six months pregnant	RED	Pillow in the shirt, confused	Yes		Yes
8	2-yo M	28	Distal pulse present	Not following commands	No	Sitting on the shoulder of the road, blood in the ears, unwilling to walk	RED	Red makeup near the ear canals, sitting, confused	Yes	Toddler size	
9	12-yo F	8	Pulseless	Unresponsive	No	Impaled by a wooden beam	RED	A wooden beam attached to the abdomen	Yes	Junior size	
10	17-yo F	0	Weak radial pulse	Unresponsive	No	Trapped under rubble, apneic after five rescue breaths	BLACK	Buried under rocks/blocks	Yes	Adult size	
11	62-yo M	28	Cap refill <2 sec	Crying for help, able to recall events	No	Leg caught under rubble, open fracture	YELLOW	Right lower leg under rubble/rocks/objects. Lifting of the leg reveals a moulaged leg with exposed bone	Yes	Adult size	Yes
12	6-mo M	0	Pulseless	Moaning initially, now unresponsive	No	Was found down on the ground with abrasions all over the body, a large occipital hematoma	BLACK	Bruising on the body, bruising on the occipital area	Yes	Infant size	
13	8-yo F	40	Weak radial pulse	Responds to verbal stimuli	No	Large bruise forming on the abdomen, abrasions on extremities, unable to walk	YELLOW	Large bruising on the stomach that is covered by the shirt, and abrasions on the extremities. The patient is holding the abdomen	Yes	Junior size	Yes
14	13-yo M	48	Rapid and weak	Blank stare	No	Partial amputation of the right arm, diaphoretic	RED	Moulaged arm (big red and black wedge on the right forearm with visible bone)	Yes	Junior size	
15	3-yo F	36	Bounding pulse	Alert but won’t speak	No	Abrasions to neck/torso, lacerations on arms with some embedded glass, refusing to move legs	YELLOW	Abrasions on the neck and torso, glass attached to the arms	Yes	Junior size	

Appendix 5 

Appendix 6 

Appendix 7

Appendix 8 

 Appendix 9 

Appendix 10 

Appendix 11  

Appendix 12 

Appendix 13  

Appendix 14 

Appendix 15

Appendix 16
